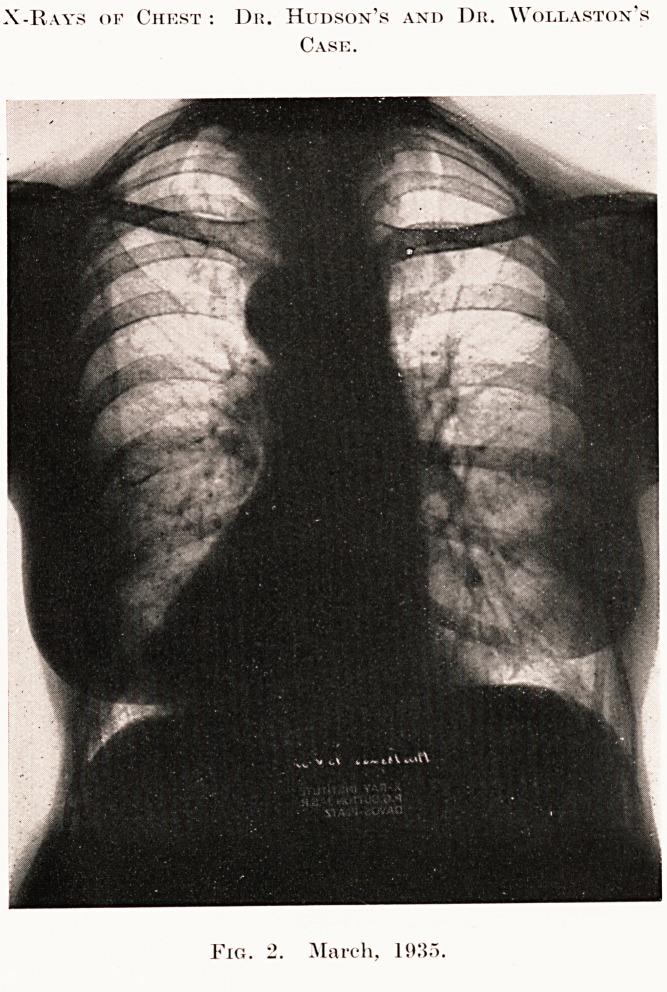# Clinical Record: A Case of Pulmonary Consolidation of Doubtful Origin

**Published:** 1935

**Authors:** B. Hudson, F. L. Wollaston

**Affiliations:** Medical Superintendent, Victoria Sanatorium, Davos; Assistant Physician, Victoria Sanatorium, Davos


### CLINICAL RECORD: A CASE OF PULMONARY CONSOLIDATION OF DOUBTFUL ORIGIN.

BY

B. HUDSON, M.D., M.R.C.P.,
*Medical Superintendent, Victoria Sanatorium, Davos,*

AND

F. L. WOLLASTON, M.D.,
*Assistant Physician, Victoria Sanatorium, Davos.*

---

THE patient, a woman of 70, was sent out to Switzerland in March, 1935. She had had an attack of influenza in November, 1934, but no doctor was called in, and though it only lasted a week, she never felt well afterwards. At the end of December she went down to Dartmoor, where she became ill again, a few days after arrival. She developed fever and a bad cough with a considerable amount of sputum. A diagnosis of bronchitis was made. However, the cough persisted for four weeks, and she lost a great deal of weight. A skiagram was then taken. This is reproduced, and shows the upper zone of the right lung occupied by an ill-defined opacity, with numerous small translucent areas. Moreover, there is also seen thickening and scattered infiltration in the lower lobe of the left lung. A diagnosis of tuberculosis was made, although the sputum contained no tubercle bacilli.

When she arrived in Switzerland in March she was already feeling better, though she was still troubled by a cough, and had failed to regain her weight. On examination, there was marked dullness over the right upper chest, with increased breath sounds and vocal resonance. There were also a few râles in front. The left base was dull to percussion, but the breath sounds were diminished, and there was a mild pleural rub and some dry râles. The sputum was scanty, containing no tubercle bacilli. The blood sedimentation was 10/23. Her temperature never went above 99° F. (rectal), although she complained of sweating at night. A skiagram was taken a fortnight later, which is reproduced, and we were surprised to find that the right upper lobe was now completely clear, though the left lower lobe showed only slight improvement. Her general condition improved rapidly, the cough disappeared, and she had regained her original weight by the end of the month. Skiagrams were then taken every month. The right lung remained the same, and is clear. The left base also cleared up considerably and progressively, though it never became normal. Physical signs diminished, the râles almost disappearing after the first month. Her blood sedimentation became quite normal, 3/7.

This case presents several interesting diagnostic features. From the history, physical signs, and X-ray appearance we had at first no doubt that the condition was tuberculous. The X-ray shadow might perhaps have been caused by a bronchial carcinoma, but the physical signs and course of the illness were against this. An apical pneumonia may give somewhat similar radiological appearance; on the other hand, the onset was gradual, and she was never seriously ill, though an upper lobe pneumococcal pneumonia is usually very severe.

### PLATE IV

X-Rays of Chest: Dr. Hudson's and Dr. Wollaston's Case.

Fig. 1. January, 1935.

### PLATE V

X-RAYS OF CHEST: DR. HUDSON'S AND DR. WOLLASTON'S CASE.

FIG. 2. March, 1935.

The second X-ray completely upset the diagnosis. It is practically impossible for a tuberculosis of the lung of such severity to clear up completely in two months without leaving a trace of fibrosis. But the physical signs, although diminished, remained, and have not disappeared in five months.

The infiltration at the left base, in view of the displacement of the heart, is probably due to pleural thickening and fibrosis, possibly originally tuberculous, but now completely quiescent. The nature of the apical lesion, however, remains a mystery. Such rapid recovery in a person of 70 years is also very remarkable, especially considering the X-ray appearance of the first radiograph.

We would be glad to have suggestions, or to hear of any similar cases.

## Figures and Tables

**Fig. 1. f1:**
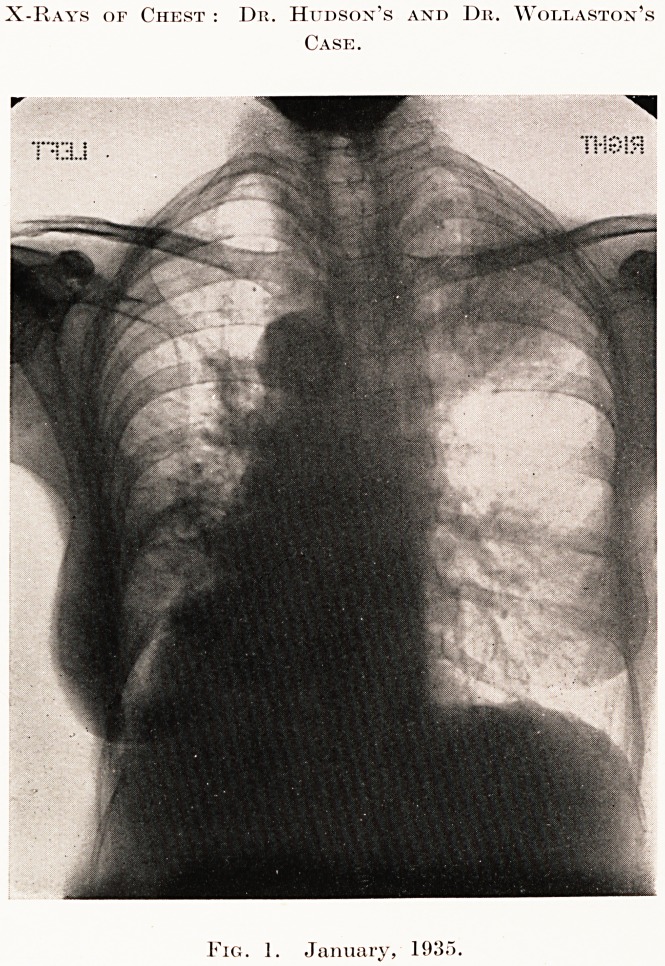


**Fig. 2. f2:**